# Nanocomposite Films of Babassu Coconut Mesocarp and Green ZnO Nanoparticles for Application in Antimicrobial Food Packaging

**DOI:** 10.3390/foods13121895

**Published:** 2024-06-16

**Authors:** Ana Carolina de Morais Mirres, Italo Rennan Sousa Vieira, Leticia Tessaro, Bruno Dutra da Silva, Jelmir Craveiro de Andrade, Arianne Aparecida da Silva, Nakédia M. F. Carvalho, Ana Maria Furtado de Sousa, Carlos Adam Conte-Junior

**Affiliations:** 1 Analytical and Molecular Laboratorial Center (CLAn), Institute of Chemistry (IQ), Federal University of Rio de Janeiro (UFRJ), Cidade Universitária, Rio de Janeiro 21941-909, RJ, Brazil; anacarolinamm4@gmail.com (A.C.d.M.M.); brunodutrads@gmail.com (B.D.d.S.); jelmirandrade@outlook.com (J.C.d.A.); conte@iq.ufrj.br (C.A.C.-J.); 2 Center for Food Analysis (NAL), Technological Development Support Laboratory (LADETEC), Federal University of Rio de Janeiro (UFRJ), Cidade Universitária, Rio de Janeiro 21941-598, RJ, Brazil; 3 Laboratory of Advanced Analysis in Biochemistry and Molecular Biology (LAABBM), Department of Biochemistry, Federal University of Rio de Janeiro (UFRJ), Cidade Universitária, Rio de Janeiro 21941-909, RJ, Brazil; 4 Institute of Chemistry (IQ), Federal University of Lavras (UFLA), Lavras 37203-202, MG, Brazil; leletessaro@hotmail.com; 5 Institute of Chemistry (IQ), Rio de Janeiro State University (UERJ), São Francisco Xavier, 524, Maracanã, Rio de Janeiro 20550-900, RJ, Brazil; arianne.a.silva@gmail.com (A.A.d.S.); nakedia@uerj.br (N.M.F.C.); ana.furtado.sousa@gmail.com (A.M.F.d.S.)

**Keywords:** antibacterial, babassu mesocarp, food preservation, nanocomposite films, natural antimicrobial, ZnO nanoparticles

## Abstract

In this work, novel nanocomposite films based on babassu coconut mesocarp and zinc oxide nanoparticles (ZnO NPs), synthesized by a green route, were produced for application as food packaging films. The films were prepared using the casting method containing different contents of ZnO NPs (0 wt%, 0.1 wt%, 0.5 wt%, and 1.0 wt%). The films were characterized by Fourier-transform infrared spectroscopy (FTIR), X-ray diffraction (XRD), thermogravimetric analysis (TGA), scanning electron microscopy (SEM), instrumental color analysis, and optical properties. The water vapor permeability (WVP) and tensile strength of films were also determined. The antimicrobial activity of the films against cooked turkey ham samples contaminated with *Staphylococcus aureus* was investigated. The results showed that incorporating ZnO NPs into babassu mesocarp matrices influenced the structure of the biopolymer chains and the color of the films. The BM/ZnO-0.5 film (0.5 wt% ZnO NPs) showed better thermal, mechanical, and WVP properties. Furthermore, the synergistic effect of babassu mesocarp and ZnO NPs in the BM/ZnO-0.5 film improved the antimicrobial properties of the material, reducing the microbial count of *S. aureus* in cooked turkey ham samples stored under refrigeration for 7 days. Thus, the films produced in this study showed promising antimicrobial packaging materials for processed foods.

## 1. Introduction

Plastic packaging represents more than 30% of the global consumption of plastic materials and predominantly relies on petroleum-derived polymers, which persist in the environment for centuries [1]. Recycling is costly due to pigmented plastics, multilayered plastics, plastic compositions, and hard-to-separate residues. Consequently, less than 10% of plastics worldwide undergo recycling, approximately 10% are incinerated, and a staggering 80% are consigned to landfills [2]. Moreover, an estimated 3% of all plastics ultimately find their way into oceans, posing significant environmental threats and jeopardizing marine ecosystems [3]. These statistics underscore the urgent need for sustainable alternatives in packaging materials to mitigate the adverse impacts of plastic pollution on the environment [4].

In particular, the food industry has a significant demand for plastic packaging, as the population of the word and urbanization lead to increased consumption of processed foods, which heavily rely on plastic materials to ensure their preservation [5]. Conventional petroleum-based polymeric materials such as polyethylene (PE), polycarbonate (PC), poly(ethylene terephthalate) (PET), poly(vinyl chloride) (PVC), polypropylene (PP), polystyrene (PS), and polyamides have been widely used for this purpose [6]. Environmental concerns regarding the production and disposal of these materials have driven the search for eco-friendly alternatives that exhibit comparable thermal, mechanical, and barrier properties [7]. Thus, using biodegradable and bio-based polymers has been a promising alternative to synthetic petroleum-derived polymers [8,9].

In this work, a biopolymer based on babassu coconut mesocarp (*Orbignya phalerata* Mart.) was chosen as the matrix for developing films [10]. Babassu mesocarp is a low-cost material obtained as a by-product of the babassu coconut, a native species in Brazil widely distributed in the northern and northeastern regions of the country, especially in the state of Maranhão [11]. It is mainly composed of starch (68.3%), but also contains fibers and amino acids (2.51%), proteins (1.54%), soluble carbohydrates (1.25%), and lipids (0.27%) [12]. Studies have shown that babassu mesocarp has antimicrobial action due to its phytochemical composition of polyphenols and phenolic compounds [13]. Furthermore, it is widely used as a dietary supplement and in folk medicine [14]. This biopolymer based on babassu mesocarp starch has been reported as an efficient matrix for producing films with excellent mechanical, thermal, morphological, and biodegradable properties [15,16,17,18]. Thus, babassu mesocarp could be an efficient alternative for producing films applied as active food packaging.

Although bio-based polymer films are considered to be promising alternatives due to their availability, reduced cost, and biodegradation capability, they face challenges such as low tensile strength and high water absorption [19]. However, these disadvantages can be overcome by incorporating nanomaterials that enhance the functional properties of the films [7]. Thus, the addition of nanoparticles to biopolymer matrices represents a promising alternative to improve film properties. Lately, zinc oxide nanoparticles (ZnO NPs) have gained prominence due to their multifunctional properties, including good optical, semiconductor, antioxidant, and antimicrobial properties, being a potential alternative for developing food packaging films [6]. Additionally, ZnO is biocompatible, non-toxic, and can be used in various biomedical and food applications [20].

On the other hand, conventional nanomaterial synthesis methods, such as chemical reduction, microemulsion, and thermal decomposition, are often harmful to the environment due to the use of severe conditions, toxic chemicals, and high production costs [21]. In contrast, green synthesis emerges as a sustainable and eco-efficient alternative, minimizing the use of toxic substances and harmful environmental residues [20]. Moreover, green synthesis methods using natural extracts and biomass residues have attracted interest recently due to the wide variety of plant species and available natural resources [22,23]. Recently, novel ZnO NPs synthesized from açaí (*Euterpe oleracea* Mart.) berry seed residue extract was successfully obtained [24]. The nanomaterial had a particle size of around 60 nm, good physical stability, and potential antimicrobial and antioxidant properties [24]. Thus, the possibility of incorporating this nanomaterial into babassu mesocarp matrices for application as food packaging was considered.

Although previous studies have highlighted the potential of babassu mesocarp matrices for film production [15,16,17,18], no study has tested this material incorporated with ZnO NPs for the preservation of real food samples. Therefore, this study aims to produce novel nanocomposite films based on babassu mesocarp and different levels of ZnO NPs for application in food packaging. The nanocomposite films were investigated using various optical, spectroscopy, thermal, and mechanical characterization techniques. Finally, the most promising film was tested against cooked turkey ham samples contaminated with *Staphylococcus aureus*, aiming for potential application as antimicrobial food packaging.

## 2. Materials and Methods

### 2.1. Materials

Glycerol and calcium chloride dihydrate (CaCl_2_·H_2_O) were purchased from Sigma-Aldrich (São Paulo, Brazil). The babassu coconut mesocarp flour was purchased from the local market in Chapadinha, state Maranhão, Brazil (3°44′26″ S and 43°21′33″ W). Before use, the material was sieved through a polyamide mesh to remove coarse particles. Zinc oxide nanoparticles (ZnO NPs) were obtained by green synthesis route using açaí (*Euterpe oleracea* Mart.) berry seed residue extract, according to our published procedure [24]. 

### 2.2. Preparation of Nanocomposite Films

The films were prepared using the two-step casting method [15]. Briefly, varying contents of ZnO NPs were dispersed in 100 mL distilled water using an Ultra-turrax^®^ homogenizer (IKA^®^ T10 basic) for 5 min and magnetic stirring at 25 °C for 60 min. Then, 1.5 g of babassu mesocarp flour and 0.5 mL of glycerol were added to the ZnO NPs suspension under continuous stirring at room temperature for 60 min. Subsequently, 1.5 mL of 10 g L^−1^ calcium chloride solution was added slowly under magnetic stirring at 70 °C for another 60 min. Finally, 30 mL of the pre-crosslinked solution was poured into Petri dishes and dried in an oven at 40 °C for 48 h. After drying, the films were de-molded and stored at room temperature for subsequent characterization and application.

**Figure 1:**
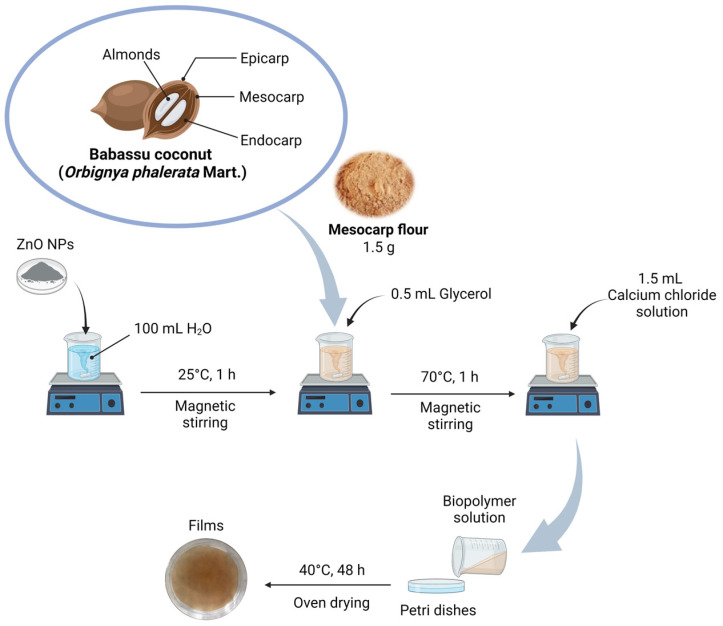
Schematic representation of the nanocomposite film preparation procedure.

The films were named as follows: BM (without ZnO NPs), BM/ZnO-0.1 (containing 0.1 wt% ZnO NPs), BM/ZnO-0.5 (containing 0.5 wt% ZnO NPs), and BM/ZnO-1.0 (containing 1.0 wt% ZnO NPs). Figure 1 shows a schematic representation of the film preparation procedure.

### 2.3. Characterization of Nanocomposite Films

#### 2.3.1. Instrumental Color Analysis

The color of the nanocomposite films was analyzed using a portable colorimeter (CM-600D Konica Minolta, Osaka, Japan). The equipment measures the reflected energy from the sample across the visible spectrum after being subjected to a light source [25]. Before the analyses, the colorimeter was calibrated using the white color of the instrument itself, and the parameters for analysis were set with illuminant D65 and a 10° viewing angle. Color measurements were taken on each film (diameter of 8 cm) at three random points on the samples. The evaluated parameters included three variables: L* = 0 (black) to 100 (white), a* = redness (+a) or greenness (−a), and b* = yellowish (+b) or bluish (−b) color [26]. These parameters were used to calculate the color saturation (C*) and hue angle (*h*°) according to Equations (1) and (2), respectively.

(1) $$C=\sqrt{a^{2}+b^{2}\;}$$

(2) $$h{}^{\circ}=\;{\mathrm{tg}}^{-1}\left(\;\frac{b}{a\;}\right)$$

#### 2.3.2. Film Thickness

The thickness of the films was measured using a digital caliper. Measurements were taken at random locations on the film, and the average thickness values were used for the calculations of opacity, water vapor permeability (WVP), and mechanical properties of the films.

#### 2.3.3. Optical Properties

The optical properties of the nanocomposite films were investigated to evaluate the UV-blocking ability through the films. Measurements were performed using a UV-Vis spectrophotometer (UV-1900i, Shimadzu, Kyoto, Japan) in the wavelength range of 200 to 800 nm. Film samples were cut into rectangular strips (45 mm × 9 mm) and placed in quartz cuvettes for light transmission analysis. An empty quartz cuvette was used as a blank simultaneously with the samples. Transmittance and opacity values were calculated using Equations (3) and (4), respectively.

(3) $$T=\frac{1}{10^{A}}$$

where *T* is the transmittance of the film samples and *A* is the absorbance.

(4) $$\mathrm{Opacity}=\frac{A_{600}}{\delta }$$

where *A*_600_ nm is the absorbance at 600 nm and *δ* (mm) is the thickness of the films.

#### 2.3.4. Fourier-Transform Infrared Spectroscopy (FTIR)

The chemical composition of the nanocomposite films was investigated using a Fourier transform infrared absorption (FTIR) spectrometer (Shimadzu^®^—IRPrestige-21, Kyoto, Japan) combined with an attenuated total reflectance (ATR) accessory with diamond crystal. The film samples (1 cm × 1 cm) were placed on the crystal cell and pressed onto the surface using a pressure anvil. FTIR spectra were acquired with 45 scans in the infrared radiation range between 4000 and 400 cm^−1^ at a speed of 0.2 cm s^−1^ and resolution of 4 cm^−1^. Readings were taken in triplicate at a controlled temperature of 23 °C. Before acquiring each spectrum, the ATR crystal was carefully cleaned with ultrapure water and subsequently dried with a tissue. The bands of the different chemical groups were identified using software and assigned using values from the literature.

#### 2.3.5. X-ray Diffraction (XRD)

The crystalline structure of the nanocomposite films was analyzed by X-ray diffraction (XRD) using a D8 Advance Bruker diffractometer. The XRD instrument was outfitted with Cu Kα radiation (λ  =  1.5406 Å), operating a voltage and current of 40 kV and 40 mA, respectively. The range of 2θ diffraction angle was 2 to 60° with a pitch of 0.02°.

#### 2.3.6. Thermogravimetric Analysis (TGA)

The thermal degradation of the nanocomposite films was measured using a thermogravimetric analyzer (TGA), model Q50 from TA Instruments, with a scanning temperature range of 50 to 800 °C and a heating rate of 10 °C min^−1^ under an inert nitrogen atmosphere with a constant flow rate of 60 mL min^−1^ of the sample. Nickel (Ni), aluminum (Al), and perkalloy standards were used for equipment calibration. The sample mass ranged from 2.0 to 4.0 mg and was analyzed in a platinum capsule.

In this analysis, the initial degradation temperatures of the films were obtained from the TGA curves by tracing the tangent line and determining the inflection point of the curve at each stage. Furthermore, the maximum points of the derived thermogravimetric (DTG) curves were assigned to the temperatures at which the degradation rate of each stage is maximum.

#### 2.3.7. Water Vapor Permeability (WVP)

The WVP test was performed using the desiccant gravimetric method according to the ASTM E96 standard. Briefly, polyethylene bottles filled ¾ of the volume with CaCl_2_ previously dried at 150 °C for 24 h were sealed with the films and placed within a chamber regulated at 23 °C and 50% relative humidity (RH) using a dehumidifier. The internal RH of the chamber remained at 0%, establishing a vapor pressure gradient facilitating water transport through the films. Mass (g) change over time (h) was tracked by weighing the system immediately after sealing, periodically over 7 h, and again after 24 h. The sampling area corresponds to the opening of the container [27]. All tests were performed in duplicate. WVP values were determined using Equation (5).

(5) $$\mathrm{WVP}=\frac{G\;\times \;\delta }{{t\;\times \;A\;\times \;S\;\times \;(R}_{1}-{\;R}_{2})}$$

where WVP is the water vapor permeability of the film [(g mm) (m^2^ s Pa)^−1^]; δ is the film thickness (mm); A is the exposed film area (m^2^); S is the water saturation vapor pressure at the test temperature (Pa); R_1_ is the external relative humidity; R_2_ is the internal relative humidity; G/t is the water permeability rate (g s^−1^), calculated by linear regression of the mass–time relationship.

#### 2.3.8. Mechanical Properties

Tensile tests were carried out on a universal testing machine (EMIC, DL2000, São Paulo, SP, Brazil) with a load cell of 100 N, claw separation speed of 500 mm min^−1^, and distance between claws of 50 mm, following the ASTM D882-12 standard adapted. A total of 7 to 9 specimens were prepared by cutting the films into rectangular sections with a length of 90.00 mm, a width of 5.00 mm, and an average cross-sectional thickness of 0.15 ± 0.06 mm. 

In this study, the values obtained for tension and elongation tests were considered as the average of at least seven determinations provided directly by the dynamometer. From the tension–elongation curves, the following properties were determined: tensile strength, elongation at break, and Young’s modulus. The latter was determined by the slope of the straight line of the tension–elongation curves where the deformation is elastic.

#### 2.3.9. Scanning Electron Microscopy (SEM)

The fracture surface of the nanocomposite films was evaluated after tensile tests using a JEOL 6510 LV scanning electron microscope (SEM). The samples were fixed to carbon tape and covered with a thin layer of gold to increase electrical conductivity. Images were obtained under high vacuum, with an acceleration voltage of 10 kV and magnifications of 200× and 700×.

### 2.4. Antimicrobial Effect of Nanocomposite Films on Cooked Turkey Ham Samples

Cooked turkey ham samples were purchased at the local market in Rio de Janeiro (Brazil) and taken immediately to the analysis laboratory. The turkey ham slices were cut into a mold with the same diameter as the film (90 mm). Based on the overall results, BM film and BM/ZnO-0.5 film were selected for antimicrobial evaluation. A control treatment (C) of turkey ham samples was used.

Strains of *Staphylococcus aureus* (ATCC 13565) were activated in Brain Heart Infusion (BHI) broth (ACUMEDIA, MI, USA) with incubation for 24 h at 35 °C. After activation, bacterial colonies were isolated on Baird–Parker Agar (KASVI, Spain) supplemented with egg yolk tellurite (MILLIPORE, Burlington, VT, USA) with incubation for another 24 h at 37 °C. Colonies of isolated bacteria were transferred to test tubes containing 5.0 mL of 0.85% (*w*/*v*) sterile saline solution. Turbidity was compared to a standard barium sulfate solution equivalent to the 0.5 MacFarland scale, corresponding to an approximate concentration of 8 log CFU mL^−1^ based on the Clinical and Laboratory Standards Institute guidelines (CLSI, 2006) [28].

The bacterial contamination assay was adapted using the method described by Dutra et al. (2022) [29]. In sterilized trays, 90 μL of a *S. aureus* suspension diluted to 6 log CFU ^−1^ was inoculated individually onto the turkey ham slices (10 g). The cultures were spread individually on the surface of the turkey ham samples with a sterile Drigalski spatula and allowed to adhere to the samples for 15 min in a bacteriological cabinet. After inoculation, the films were placed in contact with the turkey ham and the samples were stored at 7 °C.

Microbiological analyzes were performed immediately after inoculation (0D) and on days 2 (2D), 6 (6D), and 7 (7D) of refrigerated storage, aiming to simulate the average shelf life of the product, which is approximately one week under refrigeration [30]. Approximately 10 g of turkey ham samples from treatments C, BM, and BM/ZnO-0.5 were transferred to 90 mL of a 0.85% (*w*/*v*) saline solution in sterile sample bags. After homogenization in a Stomacher (Sample Mixer Stomacher, Stomax, São Paulo, Brazil) for 2 min, the solution was diluted in decimal series (10^−1^ to 10^−4^) and plated in duplicate on Baird–Parker Agar with egg yolk tellurite. The plates were incubated at 37 °C for 24 h. Results were expressed as log CFU g^−1^. *S. aureus* was also evaluated on the day of processing in samples of inoculated turkey ham slices.

### 2.5. Statistical Analysis

The experimental data were recorded as mean ± SD (standard deviation). A one-way analysis of variance (ANOVA) was executed to ascertain significant differences among treatments, followed by the Tukey post hoc test. Statistical evaluations were carried out using XLSTAT software (version 2022, Addinsoft, New York, NY, USA), where statistical significance was considered at a *p*-value (probability value) < 0.05.

## 3. Results and Discussion

### 3.1. Instrumental Color Analysis and Optical Properties

**Figure 2:**
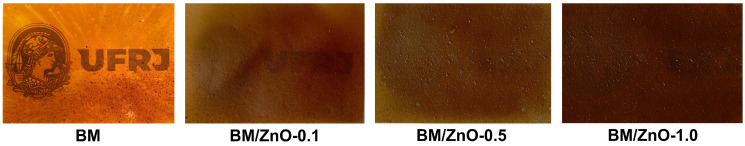
Photographs of babassu mesocarp films and their nanocomposites based on ZnO NPs. BM film (without ZnO NPs), BM/ZnO-0.1 film (containing 0.1 wt% ZnO NPs), BM/ZnO-0.5 film (containing 0.5 wt% ZnO NPs), and BM/ZnO-1.0 film (containing 1.0 wt% ZnO NPs).

Figure 2 shows the visual aspect of the films. All films produced from the babassu mesocarp were intact, flexible, and homogeneous, with a yellowish color at different opacity levels. The BM film (without ZnO NPs) appeared more translucent, while the nanocomposite films were opaque due to the presence of ZnO NPs.

The visual characteristics of the films were confirmed from color analysis using a portable colorimeter. Data related to the color parameters (L*, a*, and b*) of the films are shown in Table 1. It was observed that both the babassu mesocarp and the ZnO NPs influenced the color of the films. The films showed a yellowish color mainly due to the concentration of carotenoid pigments in the babassu mesocarp [31]. Thus, it was confirmed that the BM film was the most yellowish due to the highest value of the b* parameter (39.90), which characterizes the yellow color in the color spectrum. The yellow color of babassu mesocarp pigments has also been previously reported [18].

The films also exhibited redness, according to the positive parameter a*. Additionally, it was observed that the addition of ZnO NPs decreased the values of luminosity (L*) [26], with the BM/ZnO-1.0 film being the darkest, with L* = 33.77. As for color saturation, it was higher for the BM film with C* = 42.84, whereas the BM/ZnO-1.0 film appeared to be less opaque and had lower color saturation (C* = 24.77). The MB/ZnO-0.1 (55.83°) and MB/ZnO-0.5 (55.58°) nanocomposite films did not show significant differences in hue angle values (*h*°), with colors closer to red about the MB film (68.67°) and MB/ZnO-1.0 film (60.22°), which came closest to the yellowish color [32,33].

**Figure 3:**
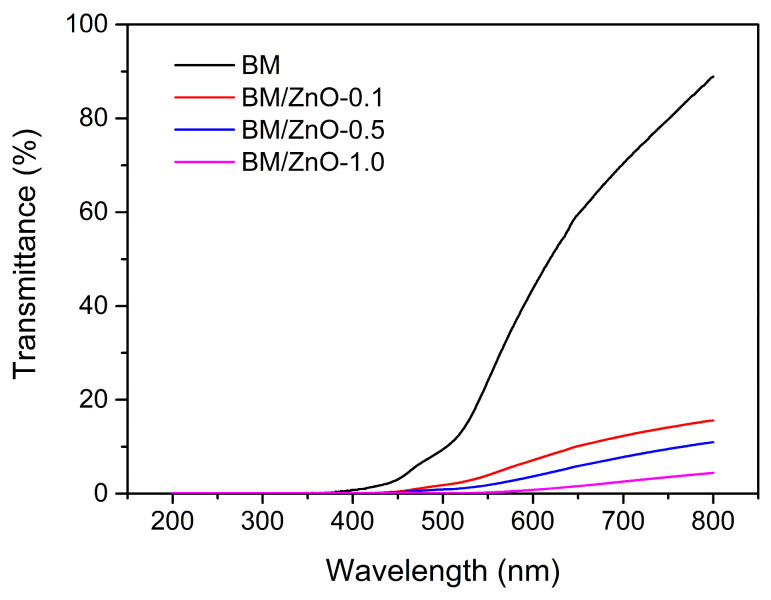
UV–Vis absorption spectra of the films. BM film (without ZnO NPs), BM/ZnO-0.1 film (containing 0.1 wt% ZnO NPs), BM/ZnO-0.5 film (containing 0.5 wt% ZnO NPs), and BM/ZnO-1.0 film (containing 1.0 wt% ZnO NPs).

UV-Vis absorption spectra ranging from 200 to 800 nm are presented in Figure 3. The opacity of the films increased with the addition of ZnO NPs. The BM film obtained the lowest opacity value among the films produced (10.62), while the BM/ZnO-0.5 nanocomposite was the opaquest (21.52). The BM/ZnO-1.0 nanocomposite had the lowest opacity among the nanocomposite films (12.01); this effect may be related to the compatibility of the ZnO NPs with the polymer matrix and the greater thickness of the film (0.259 mm) [34]. The other film samples presented average thicknesses ranging from 0.113 to 0.128 mm, without significant differences between the samples (*p* > 0.05).

In summary, incorporating ZnO NPs increased the resistance of the films to UV light and opacity but decreased its transparency [35]. Therefore, the nanocomposite films produced could be helpful as packaging for foods prone to oxidative degradation caused by exposure to UV light. Similar results were previously reported on the optical properties of starch packaging films reinforced with ZnO NPs [36].

### 3.2. FTIR and XRD Analysis

The chemical composition and crystalline structure of the nanocomposite films were investigated by FTIR and XRD analyses, respectively [37]. 

**Figure 4:**
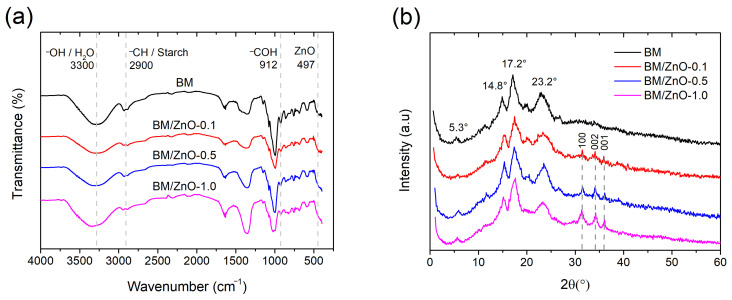
(**a**) FTIR spectra and (**b**) XRD patterns of nanocomposite films based on babassu mesocarp and ZnO NPs. BM film (without ZnO NPs), BM/ZnO-0.1 film (containing 0.1 wt% ZnO NPs), BM/ZnO-0.5 film (containing 0.5 wt% ZnO NPs), and BM/ZnO-1.0 film (containing 1.0 wt% ZnO NPs).

Figure 4a shows the FTIR spectra of the films. In general, the films showed vibration of the –CH groups at 2900 cm^−1^, which can be attributed to the starch bonds in the babassu mesocarp [38]. The –OH groups of water absorbed around 3300 cm^−1^, while the –COH bond present in starch was observed at frequencies of 912 cm^−1^ [18]. Other vibrations were observed in the regions between 1012 cm^−1^ and 1164 cm^−1^, attributed to the C–O–C groups of polysaccharides [39]. Furthermore, the vibration range around 497 cm^−1^ is characteristic of the Zn-O bond of inorganic nanomaterial, as presented in our previous study [24].

The XRD patterns of the films are shown in Figure 4b. The results show that all films presented peaks at 2*θ* values of 5.3°, 14.8°, 17.2°, and 23.2°, characteristic of the type B polymorphic starch pattern [18]. In addition to the previous peaks, the nanocomposite films (BM/ZnO-0.1, BM/ZnO-0.5, and BM/ZnO-1.0) also presented three low-intensity peaks, corresponding to the crystalline planes of (100), (002), and (001) from the XRD pattern of ZnO NPs [24]. These peaks have been previously confirmed and correspond to the wurtzite-type hexagonal crystal structure of ZnO [24].

### 3.3. TGA Analysis

**Figure 5:**
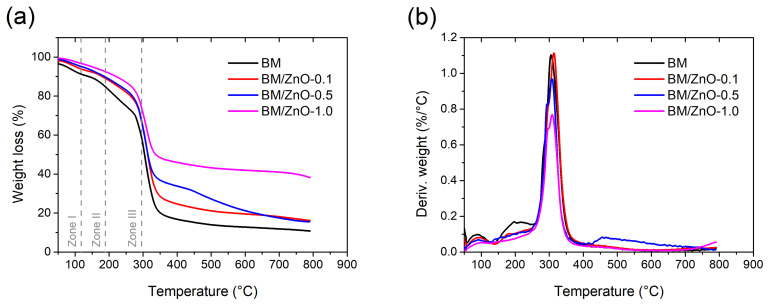
(**a**) TGA and (**b**) DTG curves of the films. BM film (without ZnO NPs), BM/ZnO-0.1 film (containing 0.1 wt% ZnO NPs), BM/ZnO-0.5 film (containing 0.5 wt% ZnO NPs), and BM/ZnO-1.0 film (containing 1.0 wt% ZnO NPs).

The thermal stability study of the films was carried out as shown in the weight loss curves (TGA) and their respective derivatives (DTG), according to Figure 5. 

The TGA and DTG curves show different stages of thermal degradation, which depended on the ZnO NPs content in the films (Figure 5). Three discrete stages of degradation were observed in the BM film (without ZnO NPs). According to Figure 5a, mass loss in the first stage (Zone I) at 68 °C was related to the release of water molecules, with a maximum degradation rate at 87 °C (Figure 5b) [40]. In this case, the moisture content of the films could influence the degradation temperature and mass loss in the first stage of degradation. Furthermore, the crystalline structure of biopolymer chains and the additives used in the preparation of the films can influence the thermal degradation of polymeric materials. The second stage (Zone II) of degradation at 165 °C can lead to the release of other low molecular weight molecules in the samples, such as remaining water fragments. In the third stage (Zone III), the degradation temperature of 283 °C may be related to the decomposition of organic compounds, including carbohydrates, lipids, proteins, and minerals [17]. Furthermore, this step is generally associated with the depolymerization of polymer chains, decarboxylation, and decomposition of glycosyl groups [41]. In addition, in this last stage, the DTG curve showed a maximum degradation temperature of 305 °C (Figure 5b).

The nanocomposite films exhibited a significant increase in thermal stability, particularly the BM/ZnO-0.5 and BM/ZnO-1.0 films, due to the incorporation of ZnO NPs. The residue at 800 °C is much higher for BM/ZnO-1.0 (around 38%), showing a considerable effect on the thermal stability of the material. On the other hand, the BM/ZnO-0.1 film showed a thermal degradation profile similar to the BM film, probably due to the low percentage of ZnO NPs (Figure 5a). Nonetheless, the initial degradation temperature increased from 68 °C (BM film) to 88 °C (BM/ZnO-0.1 film) (Figure 5a), and the maximum degradation temperature rose from 305 °C (BM film) to 315 °C (BM/ZnO-0.1 film) (Figure 5b). The other stages of degradation were considered within the error margin of equipment.

Although the BM/ZnO-0.5 and BM/ZnO-1.0 films significantly increased in the first degradation stage (274 °C and 275 °C, respectively), the values did not differ between the samples (Figure 5a). This suggests that a content of 0.5 wt% ZnO NPs may be suitable for obtaining thermally stable nanocomposite materials. Furthermore, a high percentage of residual matter was observed in the nanocomposite films due to the incorporation of inorganic nanomaterials.

Similar results were reported in the study by Abdullah et al. (2020) [41], where the TGA curves indicated that the maximum degradation temperature of nanocomposite films based on cassava starch and 2% ZnO was higher than the film without ZnO. Furthermore, Lian et al. (2021) [42] reported that corn starch and ZnO films showed mass loss temperatures above 300 °C.

### 3.4. WVP

**Figure 6:**
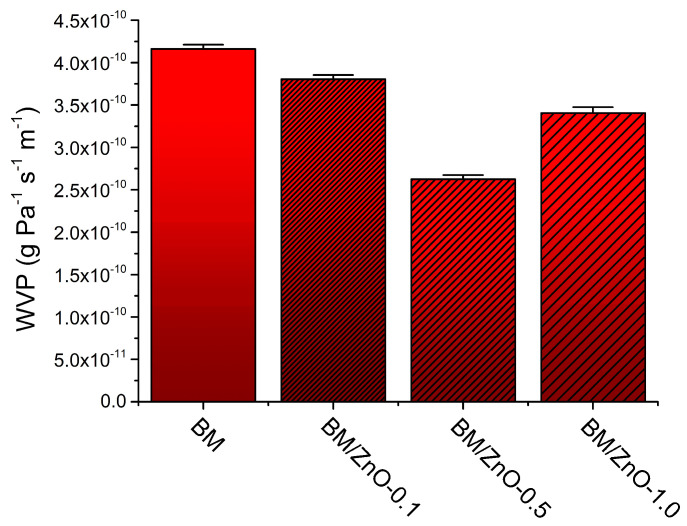
Water vapor permeability (WVP) data of the films. Error bars represent standard error. BM film (without ZnO NPs), BM/ZnO-0.1 film (containing 0.1 wt% ZnO NPs), BM/ZnO-0.5 film (containing 0.5 wt% ZnO NPs), and BM/ZnO-1.0 film (containing 1.0 wt% ZnO NPs).

The WVP test was carried out to evaluate whether nanocomposite films could be suitable for food packaging, as it allows the evaluation of the capacity of materials as a protective barrier to food. In general, biopolymer-based packaging materials can be sensitive to humidity under different atmospheres and microbial attacks, affecting the shelf life of packaged products [43]. Figure 6 shows the WVP results for the films in this study.

The WVP values of the films ranged from 4.16 × 10^−10^ g Pa^−1^ s^−1^ m^−1^ to 2.62 × 10^−10^ g Pa^−1^ s^−1^ m^−1^. This result aligns with previous studies that used different biopolymer matrices and ZnO NPs [36]. Except for the BM/ZnO-1.0 film, the increase in the content of ZnO NPs resulted in a decrease in WVP. It was observed that the BM film presented the highest WVP value (4.16 × 10^−10^ g Pa^−1^ s^−1^ m^−1^), while the BM/ZnO-0.5 film was the most efficient in the test, with a WVP value of 2.62 × 10^−10^ g Pa^−1^ s^−1^ m^−1^. This phenomenon can be attributed to the increase in moisture diffusivity resulting from the creation of interstitial gaps resulting from the aggregation of nanoparticles and the formation of a long and tortuous path for molecules within the film section [36].

On the other hand, the BM/ZnO-1.0 film showed an increase in the WVP value (3.41 × 10^−10^ g Pa^−1^ s^−1^ m^−1^) when compared to the BM/ZnO-0.5 film. The increase in the content of ZnO NPs can result in instability of the polymeric system and, consequently, the retention of more moisture in the film. Studies suggest that this phenomenon may occur due to the increase in free volume around the particles at the film interface and the increase in the tortuosity of the path taken by the molecules within the membrane, facilitating the passage of water molecules through the film [43]. This effect was demonstrated in the study by Marra et al. (2016) [43], in which poly(lactic acid) (PLA) and 1 wt% ZnO film showed an increase in the WVP value compared to pure PLA film.

### 3.5. Mechanical Properties

**Figure 7:**
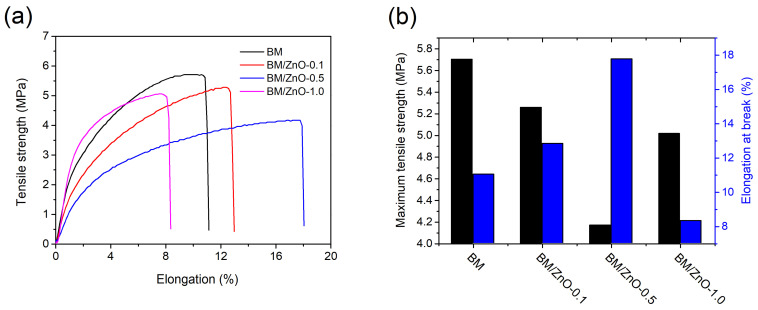
(**a**) Tensile strength–elongation curves (**b**) and data on maximum tensile at break and maximum elongation at break of the films. BM film (without ZnO NPs), BM/ZnO-0.1 film (containing 0.1 wt% ZnO NPs), BM/ZnO-0.5 film (containing 0.5 wt% ZnO NPs), and BM/ZnO-1.0 film (containing 1.0 wt% ZnO NPs).

The mechanical properties of food packaging films are significant in analyzing their strength and ability to withstand external pressure [44]. Figure 7a shows the tensile versus elongation at break curves (test specimen corresponding to the median value) of the films, while the maximum tensile strength and elongation at break values are presented in Figure 7b.

As shown in Figure 7a, the increase in the content of ZnO NPs decreased the mechanical properties of nanocomposite films. The BM film presented a tensile at break of 5.7 MPa, while the nanocomposite films presented tensile at break values between 4.2–5.3 MPa. The reduction in tensile of nanocomposite films can be attributed to the weak interactions between the nanomaterial and the polymer matrix, as well as the presence of agglomerates that can reduce the contact surface area of the polymer matrix and the nanomaterial [45]. Furthermore, the formation of hydrogen bonds between polymer chains can be disturbed due to the presence of the nanomaterial [46].

The above argument can also explain the effect of decreasing Young’s modulus with increasing ZnO NPs content, which ranged from 0.97 MPa to 2.39 MPa for nanocomposite films. On the other hand, the increased elongation of nanocomposite films can be an exciting property for food packaging since, in specific applications, such as PVC packaging films, the material needs to undergo considerable deformation to wrap the food [47]. Except for the BM/ZnO-1.0 film, the nanocomposite films showed an increase in elongation at break, ranging from 13% to 18%, which can provide a more homogeneous structure to the films. However, the MB/ZnO-1.0 film showed the lowest elongation (around 8%) compared to the other nanocomposite films, probably due to the presence of agglomerates and imperfections in the film, preventing tensile distribution throughout the sample [43,45]. These results corroborate the WVP test, and the BM/ZnO-0.5 film was chosen for the subsequent antimicrobial evaluation stage.

### 3.6. Morphological Analysis by SEM

**Figure 8:**
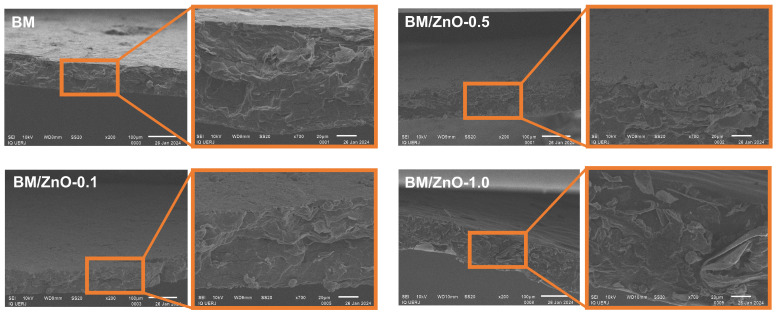
SEM images of the nanocomposite films after tensile testing in different magnifications (200× and 700×). BM film (without ZnO NPs), BM/ZnO-0.1 film (containing 0.1 wt% ZnO NPs), BM/ZnO-0.5 film (containing 0.5 wt% ZnO NPs), and BM/ZnO-1.0 film (containing 1.0 wt% ZnO NPs).

The morphology of the cross-section after fracture in the tensile test of the films is shown in Figure 8. The SEM images revealed that the films presented a morphology with rough, irregular structures, and there was no significant difference in the morphology of the films with and without the addition of ZnO NPs, suggesting compatibility of ZnO NPs with the babassu mesocarp matrix [48]. However, in the BM/ZnO-1.0 film, the roughness and cracks were more pronounced, presumably caused by ZnO clustering [36]. Excess ZnO NPs can impede the movement of macromolecular segments in nanocomposite films and disrupt their structure [42].

### 3.7. Antimicrobial Effect of Nanocomposite Films on Cooked Turkey Ham Samples

Antimicrobial food packaging is active materials containing one or more antimicrobial agents that protect against food pathogen microorganisms [6]. In this work, cooked turkey ham samples were selected for antimicrobial testing against *S. aureus* since this food matrix may be susceptible to microbial contamination even under low storage temperature conditions [49]. 

The results of the antimicrobial analysis are shown in Table 2. The control treatments (C) had no statistical difference in bacterial concentration over the days of storage (*p* > 0.05). In the BM film, the concentration of *S. aureus* was significantly reduced from the 6th day onwards and obtained a significant difference of 0.53 log CFU g^−1^ (*p* < 0.05) compared to the control treatment (C) on the same day. The treatment with the BM/ZnO-0.5 film was the one that achieved the greatest reduction in *S. aureus* compared to the control on days 6 and 7 of storage, with a significant difference of 1.04 log CFU g^−1^ and 0.71 log CFU g^−1^, respectively (*p* < 0.05). Therefore, time and treatment influenced the bacterial growth of the turkey ham samples.

According to the literature, the antimicrobial activity of babassu mesocarp may be related to the presence of bioactive compounds, especially polyphenols and phenolic compounds [13]. Several mechanisms are proposed, such as the interaction between polyphenol complexes with polysaccharides and extracellular proteins, which can cause damage to cell walls, in addition to inducing oxidative damage to DNA and inhibiting the synthesis of nucleic acids [50].

In turn, ZnO NPs synthesized from açaí seed residue extracts demonstrated broad antimicrobial activity against Gram-positive and Gram-negative bacteria, as published in our previous study [24]. This effect can be attributed to the large specific surface area and small particle size that allows them to passively pass through the bacterial cell wall and interact with intracellular components. Furthermore, other mechanisms have been proposed, such as the rupture of the bacterial phospholipid bilayer due to the electrostatic interactions of metal ions (Zn^2+^), causing extravasation of intracellular content and the production of reactive oxygen species (ROS), such as hydroxyl radicals (•OH), superoxide (O_2_^•−^) and hydroperoxyl (•OOH), which can degrade DNA, proteins, and lipids, decreasing metabolism and leading to cell death [20,24].

Previous studies have reported the antimicrobial effect of ZnO nanostructures in matrices of other biopolymers. Recently, Wang et al. (2020) [51] developed films based on chitosan, potato protein, linseed oil, and ZnO NPs to maintain the storage quality of raw meat. The results indicated that the nanocomposite film had a protective effect on raw meat during 7 days of storage, with a total bacterial (natural meat microbiota) count of around 4.5 log CFU g^−1^. This result was significantly lower than that of the control group (*p* < 0.05), above 7.5 log CFU g^−1^ on the 7th day of storage. In another study, gelatin and agar films incorporated with 1 wt% ZnO NPs were tested for storing bread. The total bacterial count values in the bread samples packaged with nanocomposite films were 4.7 log CFU g^−1^ and 4.9 log CFU g^−1^, respectively, and in the control samples, it was 6.2 log CFU g^−1^ after 9 days of storage [52].

The greater antimicrobial effect of the BM/ZnO-0.5 film in this study may be due to the synergistic contribution of the natural compounds present in the babassu mesocarp and the ZnO NPs obtained via the green synthesis route. Therefore, the nanocomposite film produced in this study could be a promising alternative for the antimicrobial packaging of processed foods.

## 4. Conclusions

Nanocomposite films based on babassu coconut mesocarp and zinc oxide nanoparticles (ZnO NPs) produced by green synthesis were successfully obtained. The incorporation of different contents of ZnO NPs influenced the structure of the biopolymer chain arrangement and the optical properties of films, altering their color and light permeability. The introduction of 0.5 wt% ZnO NPs significantly enhanced the resistance to water vapor transmission of films and elevated their thermal stability. Despite not exhibiting the highest mechanical strength, the nanocomposite film displayed remarkable elongation, providing greater flexibility and making it ideal for applications such as flexible packaging films. Moreover, the nanocomposite film demonstrated promising potential for inhibiting the microbial growth of *S. aureus* in turkey ham samples under different refrigerated storage times. Consequently, the babassu mesocarp nanocomposite film containing 0.5 wt% ZnO NPs can be a promising material for application in the processed food sector. To meet food safety requirements, future investigations could be carried out to evaluate the migration potential of ZnO nanomaterials in foods. Furthermore, in vivo cytotoxicity assays could offer a more detailed investigation of the long-term cytotoxic effects of nanomaterials.

## Figures and Tables

**Table 1:** Instrumental color and opacity data of the films.

Samples	Instrumental Color Parameters					Opacity
	L*	a*	b*	C*	*h*°	
BM	56.61 ± 0.58	15.58 ± 0.08	39.90 ± 0.03	42.84 ± 0.06	68.67 ± 0.08	10.62 ± 0.02
BM/ZnO-0.1	41.32 ± 0.69	24.03 ± 0.08	35.41 ± 0.91	42.79 ± 0.80	55.83 ± 0.60	18.65 ± 0.04
BM/ZnO-0.5	40.08 ± 0.65	20.38 ± 0.07	29.76 ± 0.75	36.07 ± 0.67	55.58 ± 0.58	21.52 ± 0.01
BM/ZnO-1.0	33.77 ± 0.98	12.29 ± 0.16	21.51 ± 1.13	24.77 ± 1.06	60.22 ± 0.98	12.01 ± 0.02

Data are expressed as mean ± standard deviation (SD). BM film (without ZnO NPs), BM/ZnO-0.1 film (containing 0.1 wt% ZnO NPs), BM/ZnO-0.5 film (containing 0.5 wt% ZnO NPs), and BM/ZnO-1.0 film (containing 1.0 wt% ZnO NPs).

**Table 2:** *S. aureus* counts (log CFU g^−1^) in cooked turkey ham samples for 7 days at 7 °C.

Treatments	0D	2D	6D	7D
C	4.93 ± 0.15 ^Aa^	4.85 ± 0.02 ^Aa^	4.68 ± 0.02 ^Aa^	4.58 ± 0.04 ^Aa^
BM	4.91 ± 0.14 ^Aa^	4.89 ± 0.16 ^Aa^	4.15 ± 0.06 ^Bb^	4.18 ± 0.03 ^Bb^
BM/ZnO-0.5	4.90 ± 0.01 ^Aa^	4.60 ± 0.15 ^Aa^	3.64 ± 0.08 ^Cb^	3.84 ± 0.20 ^Bb^

C = control treatment; BM = treatment with BM film; BM/ZnO-0.5 = treatment with BM/ZnO-0.5 film. Results expressed as mean ± standard deviation (SD), Tukey test with a significance level of 5% (*p* < 0.05). Capital letters compare the different treatments on each day of storage (columns), while lowercase letters compare the treatment during the storage period (rows).

## Data Availability

The original contributions presented in the study are included in the article, further inquiries can be directed to the corresponding author.
